# Combined Central and Peripheral Demyelination (CCPD) Associated with MOG Antibodies: Report of Four New Cases and Narrative Review of the Literature

**DOI:** 10.3390/jcm13123604

**Published:** 2024-06-20

**Authors:** Marianna Papadopoulou, Dimitrios Tzanetakos, Christos Moschovos, Anastasia Korona, George Vartzelis, Konstantinos Voudris, Stella Fanouraki, Evangelia-Makrina Dimitriadou, Georgios Papadimas, John S. Tzartos, Sotirios Giannopoulos, Georgios Tsivgoulis

**Affiliations:** 1 Second Department of Neurology, “Attikon” University Hospital, School of Medicine, National and Kapodistrian University of Athens, 12462 Athens, Greece; marpapgr@yahoo.co.uk (M.P.); dtzanetakos@med.uoa.gr (D.T.); moship@windowslive.com (C.M.); stelfanou@gmail.com (S.F.); evadim93@hotmail.gr (E.-M.D.); jtzartos@gmail.com (J.S.T.); tsivgoulisgiorg@yahoo.gr (G.T.); 2 Department of Physiotherapy, University of West Attica, Ag. Spyridonos Str., 12243 Athens, Greece; 3 Department of Neurology, Children’s Hospital of Athens “P. & A. Kyriakou”, 11527 Athens, Greece; anastasia_korona@hotmail.com (A.K.); voudrisk@gmail.com (K.V.); 4 Second Department of Pediatrics, Children’s Hospital ‘P. & A. Kyriakou’, School of Medicine, National and Kapodistrian University of Athens, 11527 Athens, Greece; vartzelius@yahoo.gr; 5 First Department of Neurology, National and Kapodistrian University of Athens, Eginition University Hospital School of Medicine, 11528 Athens, Greece; gkpapad@yahoo.gr; 6 Department of Neurology, University of Tennessee Health Science Center, Memphis, TN 38163, USA

**Keywords:** MOG, combined central and peripheral demyelination, demyelinating polyneuropathy

## Abstract

**Background/Objectives**: Myelin oligodendrocyte glycoprotein (MOG) is exclusively expressed in the central nervous system (CNS) and is found on the outer surface of oligodendrocytes. Antibodies to MOG are associated with CNS demyelination, whereas peripheral nervous system (PNS) demyelination is seldom reported to be related to MOG-IgG. **Methods**: The database of patients seen in our neurological academic center was searched for MOG-IgG seropositivity and concomitant demyelinating polyneuropathy. For the purpose of the review, in March 2024, we searched for case reports and case series in the following databases: PubMed, Scopus, Cochrane, and ScienceDirect. Inclusion criteria were MOG-IgG seropositivity and demyelinating polyneuropathy. Exclusion criteria were type of publication other than case reports and case series, unconfirmed diagnosis of demyelinating polyneuropathy, and other diseases causing demyelination in either the CNS or PNS. Critical appraisal of the selected case reports and case series was realized by JBI. **Results**: Four new cases were identified with MOG-IgG and confirmed demyelinating polyneuropathy. This review identified 22 cases that have been published since 2018. Clinical, imaging, neurophysiological, and immunological characteristics, as well as treatment options and outcomes are presented and compared to those of other cases with combined central and peripheral demyelination (CCPD). **Conclusions**: The pathogenetic mechanism is unclear; thus, different hypotheses are discussed. New case reporting and large cohort studies will help further the exploration of the underlying mechanism and guide more effective therapeutic interventions.

## 1. Introduction

Demyelinating diseases are usually immune-mediated disorders affecting either the central nervous system (CNS) or the peripheral nervous system (PNS) myelin. Because the myelin sheath differs in the CNS and the PNS, antibodies recognize and affect different antigens in the central and peripheral myelin. Demyelination affects saltatory electrical conduction from one node of Ranvier to the next, producing a delay in signal transmission, resulting in the malfunctioning of neurons. In acute attacks, symptoms might be reversible mainly due to the subsidence of inflammation. In chronic stages, remyelination may occur, but it is slower and partial. Residual symptoms reflect the persistent demyelination and secondary axonal loss [1].

Demyelinating disorders of the CNS are characterized as primary idiopathic, mostly inflammatory, and secondary, due to infections and ischemic, metabolic, or toxic causes. MRI is the examination of choice to diagnose central demyelination. Idiopathic inflammatory demyelinating diseases (IDDs) encompass a large spectrum of either monophasic, relapsing, or progressive disorders. Fulminant forms also exist and are characterized by acute and severe clinical course with atypical MRI findings. Some IDDs are disseminated in the brain and spinal cord, while others present with more restricted distribution, affecting predominantly the optic nerves and spinal cord [1].

Multiple sclerosis (MS) is the most common IDD, with a prevalence that varies considerably, from high rates in North America and Europe (>100/100,000) to low rates in Eastern Asia and Sub-Saharan Africa (2/100,000) [2]. The increasing risk of developing MS with higher and lower latitudes has been confirmed by many epidemiologists following the work of Kurtzke. The incidence of MS is two or three times higher in women than in men, while the incidence in children is considered extremely rare. The peak of the first occurrence is in the 3rd and 4th decades, falling afterward and becoming low in the 6th decade [3,4].

The etiology of the disease remains unclear. The peculiar geographical distribution has given rise to discussion regarding environmental factors, such as sun exposure and vitamin D insufficiency [5]. Hereditary factors may also play a role. A familial predisposition to MS is well established, because the risk of developing MS due to genetic factors is estimated to be 50%. It is found that 12.5% of all MS cases are familial [6]. Genetic susceptibility to MS is mainly associated with genes located in the major histocompatibility complex (MHC), and, in particular, the HLA class I and class II [7]. So, it is generally accepted that MS is a multifactorial disorder and the interaction between environmental and genetic factors may affect susceptibility to the disease.

A triggering factor has been thoroughly researched over the years that could account for the alteration in humoral and cell-mediated immunity. Until now, no virus or bacterial agent has been proven to be implicated in the pathogenesis of MS. Antibodies to specific myelin proteins (MBPs) have been identified. It is believed that exposure to foreign antigens may activate cross-reactive T-cells in genetically susceptible individuals, with MBPs being a possible autoantigen and thus it is generally accepted that MS is mainly caused by a T cell-mediated response, as a result of sensitization to a myelin protein [8]. Furthermore, humoral immunity implication can be supported by the fact that oligoclonal immune protein antibodies are present in cerebrospinal fluid (CSF) produced by B lymphocytes within the CNS. Other autoantibodies have been inconsistently detected in MS patients. Recent studies suggest that some of these cases with autoantibody-mediated demyelination should be regarded as distinct diseases such as neuromyelitis optica spectrum disorder or myelin oligodendrocyte glycoprotein antibody-associated disease (MOGAD) [9].

The diagnosis of MS is based on the evidence of dissemination in space and in time. The revision of Mc Donald’s criteria in 2017 focused on the distinction of MS from other demyelinating diseases of the CNS and incorporated new MRI criteria, CSF, and other paraclinical tests to facilitate an earlier diagnosis of MS [10].

Neuromyelitis optica spectrum disorder (NMOSD) is an autoimmune CNS astrocytopathy, with secondary demyelination, distinct from MS; based on the presence or absence of aquaporin-4 (AQP4) IgGs in the serum, patients with the NMOSD phenotype are categorized as seropositive and seronegative, respectively [11,12]. NMOSD clinical attacks are more commonly manifested with optic neuritis, myelitis, and brainstem symptoms and can be associated with severe disability accrual [12,13]

NMOSD has a worldwide distribution and an estimated prevalence of 1–5/100,000. The average annual incidence is 1/770,000 worldwide. Nearly 90% of affected individuals are female with an onset typically in late middle age [14].

In patients with acquired CNS demyelinating syndromes that are distinct from MS and NMOSD, serum antibodies directed against myelin oligodendrocyte glycoprotein (MOG) have been detected. MOG antibody-associated disease (MOGAD) is most often associated with acute disseminated encephalomyelitis, optic neuritis, or transverse myelitis and is less commonly associated with other cerebral lesions (cortical, brainstem, and cerebellar). MOGAD may have a monophasic or relapsing course, and MOG-IgG cell-based assays are important for diagnosis. The exact prevalence of MOGAD is not known because MOG-IgG was discovered in 2007 and was not widely known for many years. The incidence in nationwide studies was found to be between 1.6 and 3.4 per 1,000,000 person-years. The median age of MOGAD onset is 20 to 30 years [15,16,17].

Demyelinating disorders of the PNS may represent either as acute or chronic syndromes. Chronic inflammatory demyelinating polyradiculoneuropathy (CIDP) is an immune-mediated polyneuropathy marked by nerve roots and peripheral nerve inflammation, resulting in segmental demyelination and is the most common immune-mediated neuropathy. CIDP has a relapsing–remitting or progressive course of symmetric weakness of the proximal and distal muscles with a prevalence of 0.7 to 10.3 cases per 100,000 people [18,19]. The cause of CIDP is unknown, but cell and humoral immunity is involved. They act synergistically, but their relative contributions remain largely undefined. The target antigens are undefined, but there is evidence of antibody responses to Schwann cells, compact myelin, and nodal antigens. Moreover, the trigger for the autoimmune response has been identified. The autoimmune etiology is supported by the efficacy of treatments. A combination of autopsy, MRI, and ultrasound studies have demonstrated that the inflammatory lesions in CIDP occur predominantly in the spinal roots, proximal nerve trunks, and major plexuses [20].

Because of the lack of disease-specific diagnostic biomarkers, the diagnosis depends on the combination of clinical, electrodiagnostic, and laboratory/neuroimaging findings [21]. Criteria proposed for research practice may exclude non-typical cases. Thus, although patients may not meet the diagnostic criteria for inclusion in clinical trials of CIDP, they should be considered as suffering from CIDP and benefit from treatments. Supportive criteria for CIPD diagnosis for patients not fulfilling electrodiagnostic criteria are the response to treatment, imaging CSF, and nerve biopsy findings. 

Acute demyelinating peripheral neuropathy (AIDP) is an immune-mediated disorder with a median incidence of 1.3 cases/100,000 people [22]. Different electrodiagnostic criteria have been proposed to diagnose AIDP and to differentiate primary demyelinating from primary axonal subtypes [23]. Required features for the diagnosis of AIDP are progressive weakness of the arms and legs, absent or decreased tendon reflexes, and progressive worsening for no more than 4 weeks. The diagnosis is supported by increased protein levels in the CSF, anti-GQ1b antibodies (Abs) in Miller–Fisher Syndrome, and electrodiagnostic criteria [24].

The hallmark of the diagnosis of both CIDP and AIDP is the evidence of conduction slowing, as demonstrated by nerve conduction studies: prolongation of distal motor latency, reduction in motor conduction velocity, prolongation or absence of F-wave latency, motor conduction block, and abnormal temporal dispersion [21]. 

Combined central and peripheral demyelination (CCPD) is rare, and the relevant literature is limited to case reports and small case series [25,26]. There is no definition or diagnostic criteria to describe this unique condition. It is not clear whether the concurrent demyelination of the CNS and PNS is accidental or linked to a common shared immunopathogenic mechanism or to a higher susceptibility to autoimmune disease [26]. The most common symptoms are motor weakness, hyporeflexia, and sphincter disturbance along with optic neuritis [27]. Most of these patients present with a relapsing or progressive disease course [25], and usually CNS involvement precedes PNS involvement [26]. An example of CNS and PNS involvement is hypothesized in the case of Bickerstaff brainstem encephalitis and Miller–Fisher syndrome, because both share common clinical features and the presence of common autoantibodies, supporting the notion of a common etiology and representing a continuous spectrum rather than two different clinical entities [28].

Ιt is suggested that T-cells and autoantibodies that are involved may cross the blood–brain barrier and disseminate inflammation from one chamber to the other. Although PNS and CNS myelin are different because they are produced by different cells, oligodendrocytes in the CNS and Schwann cells in the PNS, it is known that the majority of PNS myelin proteins are also present in the CNS; e.g., P1 in the PNS is identical to BMP in the CNS [29].

Taking into account the prevalence of the two most common demyelinating diseases of the CNS and PNS, MS and CIDP, the overall report of patients with concurrent central and peripheral demyelination is rare. Evidence of demyelinating lesions of the CNS is unlikely in typical CIDP, and vice versa, no PNS involvement is present in cases of MS, on clinical grounds. Thus, the scarce reports of isolated cases support the hypothesis that combined demyelination of the CNS and PNS represents a distinct clinical entity and not an accidental concurrence of the two demyelinating diseases. This is further supported by the fact that clinical, CSF, and MRI features in these patients were atypical for MS [26].

The myelin oligodendrocyte glycoprotein (MOG) is located within the external surface of CNS myelin sheaths and the plasma membranes of oligodendrocytes [30,31]. Because of its external location, MOG serves as an accessible antigen target for autoantibodies. Cell-based assays (CBAs) have enabled the detection of antibodies against MOG in patients with MOGAD that include central nervous system (CNS) demyelinating syndromes, optic neuritis (ON), and transverse myelitis (TM) [32].

Antibodies against neurofascin-155 (NF155), a protein expressed on both central and peripheral myelin, was identified in Japanese patients with CCPD in 2013 [33]. Anti-aquaporin-4 (AQP4) antibodies and less commonly for anti-MAG (myelin-associated glycoprotein) have also been reported to be associated with CCPD [27].

Although MOG is thought to be exclusively expressed in the CNS, rare reports of PNS involvement in MOG-positive patients have appeared in the literature, the first in 2018 [34], and since then, few case reports have emerged. We present a case series of four MOG-IgG seropositive patients who presented with concurrent demyelinating peripheral neuropathy and also a review of the relevant literature.

The aim of this review is to identify all published CCPD cases with MOG-positive antibodies; describe their epidemiological, clinical, and laboratory findings; and compare them to those of the rest of CCPD cases.

## 2. Materials and Methods

### 2.1. Case Presentation

From 2016 to 2024, in the Second Department of Neurology of Attikon University Hospital, four cases were identified with MOG-IgG seropositivity and the diagnosis of demyelinating polyneuropathy, fulfilling the EFNS/PNS electrodiagnostic criteria for chronic inflammatory demyelinating polyneuropathy (CIDP) [21].

### 2.2. Review of the Literature

The protocol of this overview can be found on OSF.IO (DOI 10.17605/OSF.IO/3EKRP).

A comprehensive literature search was performed in March 2024 and included papers published after 2000 in PubMed, Scopus, Cochrane, and ScienceDirect. The literature search was composed of the Medical Subject Headings (MeSH) and free-text words for ((anti mog) OR (Myelin-Oligodendrocyte Glycoprotein)) AND (((((((((Polyneuropathy) OR (peripheral nervous system) OR (CIDP) OR (inflammatory demyelinating polyneuropathy) OR (radiculopathy) OR (AIDP) OR (Motor conduction block) OR (MMN) OR (Guillain Barre)))))))) and was implemented for different databases.

The reference lists of all the appraised articles were screened for relevant citations that might have been missed from the electronic searches. Once all articles were found, duplicate articles were removed. Two reviewers (M.P. and D.T.), with long clinical and academic experience in the diagnosis and treatment of patients with polyneuropathy and demyelinating diseases, independently screened the titles and abstracts for eligibility and examined the full text of the articles to reach a final decision. This article is presented according to the Narrative Review Reporting Checklist, available as a Supplementary File [35,36].

### 2.3. Inclusion Criteria

1. Case reports and case series were included.
2. MOG antibody (IgG) seropositivity and demyelinating polyneuropathy, following diagnostic, clinical, and neurophysiological criteria, of the Joint Task Force of the EFNS and the PNS [21], without restrictions in terms of age, sex, stage, and duration of the disease.

### 2.4. Exclusion Criteria

1. Reviews (systematic or other) and meta-analyses, clinical trials (randomized or not).
2. Animal studies.
3. Demyelination of the CNS not attributable to MOG antibodies, e.g., multiple sclerosis (MS) and seropositive aquaporin-4 neuromyelitis optica spectrum disorder (NMOSD).
4. PNS involvement did not fulfill demyelinating electrodiagnostic criteria (e.g., axonal neuropathy, migrant sensory neuritis (Wartenberg neuritis), and pain/paresthesia without clear characterization.
5. Secondary demyelinating diseases, such as infectious diseases (e.g., HIV), other inflammatory diseases (e.g., sarcoidosis), metabolic or toxic diseases (e.g., alcoholism), and inherited diseases (e.g., Charcot–Marie–Tooth)

### 2.5. Data Extraction

Both reviewers extracted data from 15 eligible studies which included authors; date of publication; number of subjects; epidemiologic data (age and sex); clinical presentation; antibody screening; CSF, MRI and nerve conduction study (NCS) findings; treatment applied; and outcome. The demographic, clinical, neurophysiological, imaging, and immunological characteristics as well as treatment and response to treatment of the four new patients and the included studies are presented in Table 1.

### 2.6. Risk of Bias

Critical appraisal of the selected case reports and case series was realized by JBI [50,51] (Supplementary File).

## 3. Results

### 3.1. Case Presentation

All patients were examined by neurologists of the Second Department of Neurology of Attikon University Hospital and specialists in demyelinating disorders of the CNS and in neurophysiology. For the diagnosis of CCPD, the inclusion and exclusion clinical and neurophysiological criteria proposed by Ogata et al. were used [52].

- **Case A:** A 20-year-old (September 1993) man, developed progressive weakness of the lower limbs and a T10 sensory level with hypoesthesia to heat, and numbness resulting, over 30 days, with difficulty standing and walking. Six months later, he was admitted to the hospital for further investigation; on examination, lower limb hypopallesthesia and spastic–ataxic gait, with downgoing plantar responses, were also noted. However, clinical findings in favor of PNS involvement were noted, i.e., diminished deep tendon reflexes in the upper limbs and almost absent in the lower limbs and mild distal lower limb weakness as well. CSF was unremarkable, with no cells, normal albumin levels, and presence of oligoclonal bands (OCBs). The MRI revealed brain lesions supratentorially, in the brainstem and cerebellum; multiple cervical and thoracic lesions, one longitudinal lesion (T6–T10 levels), with no gadolinium enhancement. Serum screening for systemic rheumatological disease, including anti-nuclear antibodies (ANAs) and antibodies to extractable nuclear antigens (anti-ENAs), was negative; complement C3 and C4 levels and vitamin B12 levels were within normal range. Although there was a strong suspicion of a diagnosis of MS, the signs of peripheral involvement led to an NCS examination that revealed demyelinating polyneuropathy (CIDP) according to EFNS/PNS electrodiagnostic criteria. Consequently, the diagnosis of CCPD was made, and genetic testing for Charcot–Marie–Tooth (CMT) disease, including CMT1-A and CMT-X subtypes, was negative. Due to a history of right optic neuritis (ON) 5 years before (1988), visual evoked potentials (VEPs) were performed showing a prolongation of P100 on right stimulation. He was treated with dexamethasone intramuscularly (8 mg × 5 days) for an MS attack and recovered fully after 40 days with mild residual numbness in his legs. After 6 months (November 1994), he developed dysesthesias and urinary incontinence and was subsequently treated with intravenous methylprednisolone (IVMP) (1 g × 5 days) and prednisolone p.o. (20 mg × 20 days) without substantial improvement; new lesions were found in a brain MRI. In the following years, the patient was treated by different physicians. During the following 8 years, he reported four clinical attacks that were treated with IVMP. He consecutively received several immunotherapies for MS, namely, intramuscular interferon beta-1a (30 mcg qWk) for 7 years that was stopped due to a clinical relapse with significant lower limb numbness; then, intravenous immune globulin pulse therapy (IVIG, 2 g/kg every 3 months) was administered for 5 years with no clinical response; then, azathioprine (50 mg TID) was tried for 1 year. Due to gastrointestinal side effects, azathioprine was switched to methotrexate (7.5 mg/week) for 1.5 years with additional clinical deterioration; gradually, he developed weakness and dysesthesia in his hands and legs, dysesthesia, and urinary incontinence. At that time, the causes of PNS involvement were further investigated; therefore, further serum screening was performed for neurofascin-155 and contactin-1 antibodies that tested negative; serum MOG antibodies were positive (1/80). A nerve biopsy was not performed, because the CCPD diagnosis was attributed consequently to MOG IgG positivity, and thus anti-B-cell therapy was initiated. Over the last 4 years, no relapses and clinical improvement have been reported under iv rituximab (600 mg every 6 months), and at the latest follow-up examination, the patient had normal sensory examination, mild distal limb weakness, and residual spastic–ataxic gait, requiring a walking aid. The timeline of the disease course is presented in Figure S1 (Supplementary Materials).
- **Case B:** A 67-year-old (2023) man, developed gradually, over 6 months, numbness in the upper and lower limbs, distal muscle weakness in the upper and lower limbs with a 4/5 score on the British Medical Research Council (BMRC) scale, lower limb hypopallesthesia (3/8 grade according to the graduated Rydel–Seiffer tuning fork)*,* absent tendon reflexes in his lower limbs, ataxic gait with instability and falls, and hoarseness. The NCS findings were compatible with demyelinating neuropathy (CIDP) according to the EFNS/PNS electrodiagnostic criteria. A brain MRI showed supratentorial, non-enhancing, subcortical, and deep white matter brain lesions, whereas the spinal MRI was negative. CSF had increased albumin and negative OCBs. Accordingly, the diagnosis of CCPD was reached. A serum autoantibody screening for autoimmune CNS inflammatory disorders including ANAs, anti-ENAs, anti-double-stranded DNA (anti-dsDNA) antibodies, C3 and C4 levels, and anti-cardiolipin and anti-β-2 glycoprotein I (anti-β2GPI) antibodies was negative, as was the screening for infectious causes, including human immunodeficiency virus (HIV). Moreover, due to PNS involvement, further laboratory investigation was performed including serum antibodies against gangliosides and a paraneoplastic antibody panel, which were negative. Monoclonal gammopathy was also investigated and excluded. In addition, the laboratory investigation of CCPD also included MOG antibodies in the serum, which tested positive. A nerve biopsy was not required because CCPD was linked to MOGAD. He received IVMP (1 g/24 h) for 3 days with a good clinical response regarding muscle weakness (BMRC score 5/5); after a 13-month treatment period with p.o. methylprednisolone (16 mg/24 h slowly tapering to 4 mg/24 h), the neurological examination showed further improvement of the lower limb hypopallesthesia (4/8 Rydel–Seiffer score) and of the gait ataxia with only mild difficulty in tandem walking The timeline of the disease course is depicted in Figure S2 (Supplementary Materials).
- **Case C:** A 10-year-old (2018) boy, presented with over 10 days of progressive muscle weakness in the lower limbs, resulting in a 3/5 score on the BMRC scale, areflexia in the lower limbs, gait instability, and incontinence. A brain MRI showed multiple supratentorial, brainstem, and cerebellar non-enhancing lesions; non-active lesions were also found in the thoracic and lower cervical spine, with thickening and enhancement of the lumbosacral roots. NCS was consistent with demyelinating polyneuropathy (CIDP) according to EFNS/PNS electrodiagnostic criteria. The CSF revealed pleocytosis (lymphocytosis) and elevated albumin with negative OCBs. There were no findings of rheumatic disease autoantibodies in the serum (ANAs, anti-ENAs, anti-ds DNA, anti-cardiolipin, anti-β2GPI, and C3 and C4 levels); infectious causes of myelitis in the CSF including herpes simplex virus 1 (HSV-1) HSV-2), human herpes virus 6 (HHV-6), varicella zoster virus (VZV), cytomegalovirus, enterovirus, West Nile virus (WNV), Epstein–Barr virus (EBV), and HIV were also absent. Anti-ganglioside antibodies associated with autoimmune peripheral neuropathies were also investigated and were negative. NMOSD antibody serum screening for MOG IgGs was positive, and anti-AQP4 was negative. The patient was diagnosed with CCPD due to MOG antibodies, and therefore a nerve biopsy was not performed. He was treated with IVMP (1 g/24 h) for 5 days followed by a 5-day course of IVIG 2 g/kg with marked improvement during the first week, and he continued on oral corticosteroids. After 3 months, an MRI was unremarkable for new lesions, but after 9 months, new lesions were observed in the thoracic spine; after 13 months, visual acuity was diminished, compatible with optic neuritis. He received IVMP (1 g × 5 days) with marked improvement and was started on preventive therapy for MOGAD with rituximab (375 mg/m^2^ per week); due to an allergic reaction during the third infusion, rituximab was switched to p.o. mycophenolate mofetil (2 g/24 h), and since then (4.5 years), he has remained in a stable condition. The timeline of the disease course is presented in Figure S3 (Supplementary Materials).
- **Case D:** A 20-year-old (February 2022) woman, reported numbness in four limbs and muscle weakness in left limbs, beginning 2 weeks before admission. On examination, she had difficulty walking, left hemiplegia, “stocking-glove” distribution hypoesthesia with abolished ankle reflexes, and lower limb hypopallesthesia (4/8 grade according to the graduated Rydel–Seiffer tuning fork). An MRI of the brain and spine revealed multiple T2-weighted hyperintense lesions with no gadolinium enhancement; in detail, few for MS non-typical brain lesions and multiple spinal lesions at the C1–C2 levels with a central location on the axial plane, C3 level, and T5 and T8 levels. Due to the distribution of the aforementioned sensory symptoms, the patient was investigated for polyneuropathy NCS-confirmed demyelinating polyneuropathy (CIDP) according to the EFNS/PNS electrodiagnostic criteria. CSF showed elevated albumin and OCBs type II. Serum and CSF screening for infectious causes of myelitis (WNV, EBV, HIV, HSV-1, HSV-2, HHV-6, VZV, cytomegalovirus, and enterovirus) and serological testing for autoantibodies in systemic autoimmune diseases (ANAs, anti-ENAs, anti-ds DNA, anti-cardiolipin, anti-β2GPI, and C3 and C4 levels) were normal. In the context of atypical CNS demyelination, serum MOG antibodies tested positive, whereas anti-AQP4 tested negative, suggesting the diagnosis of MOGAD with CCPD; therefore, a nerve biopsy was not performed. She was treated with IVMP (1 g/24 h) for 5 days followed by p.o. corticosteroids (methylprednisolone), starting dose 64mg/day; the numbness subsided, and ankle reflexes and muscle strength and gait were restored over the next 2 weeks. Four months (June 2022) after the clinical attack, she had only residual left-hand numbness; on examination, there was lower-limb hypopallesthesia with normal muscle strength. The patient refused prophylactic treatment. However, 26 months after the first episode (April 2024), she relapsed with left hemiparesis and two new enhancing lesions in the brain and cervical MRI. The patient received IVMP (1 g × 5 days) with a favorable clinical response (resolution of hemiparesis) and was switched to long-term immunotherapy; she received the starting dose of iv rituximab (600 mg weekly for 4 weeks, May 2024), and subsequently, follow-up infusions (600 mg) have been planned for every 6 months. The timeline of the disease course is depicted in Figure S4 (Supplementary Materials).

### 3.2. Review of the Literature

A total of 19 patients were identified as positive for MOG IgG and for central and peripheral demyelination; 11 derived from single case reports (patients 9–22) [34,39,40,41,42,45,46,47,48,49] and 8 from two case series (patients 1–8) [37,38]. There were three cases of anti-MOG-positive demyelinating polyneuropathy without evidence of CNS involvement (patients 14–16) [43,44]. The first case report was published in 2018 (patient 9) [34]. 

Ten out of twenty-two patients were male (45%). The mean age was 40 (7–74), four of the patients being children (18%) (patients 3, 16, 21, and 22). Seven out of twenty-two (32%) patients had a history of ON (patients 2, 4, 6, 7, 11, 19, and 21). Ten out of twenty-two (45%) patients had spinal cord lesions (patients 1, 3, 5, 8, 9, 10, 12, 13, 17, and 20) [34,37,38,39,42,45,48]. In two cases, patients presented with both ON and myelitis (patients 18 and 22) [46,49], while there were three cases with only PNS involvement without clear CNS involvement (neither myelitis nor ON) (patients 14–16) [43,44]. 

The presentation of CNS and PNS involvement was concurrent in 10 cases (45%) (patients 3, 5, 6, 9,11–13, 17, 20, and 22); ON preceded in 5 cases (patients 2, 7, 18, 19, and 21) (23%), myelitis in 2 cases (0.1%) (patients 1 and 10), and PNS in 2 cases (patients 4 and 8) (0.1%) [38], one being multifocal motor neuropathy (MMN) and the other brachial neuritis. Regarding past medical history, two cases occurred 3 months post-partum, (patients 5 and 13), two cases were after a SARS-CoV-2 infection (patients 16 and 19), one case was after an influenza-A infection (patient 12) [42], and one case occurred 6 days after an upper respiratory tract infection (patient 22). One case occurred 2 weeks after an influenza-A H1N1 vaccination (patient 8). In three cases, it was clearly described that no preceding infection or vaccination (patient 9, 14, and 15) was recorded. For the rest of the patients, there is no specific information provided; in one case, an episode of ON 20 years before is reported (patient 11).

The clinical presentation of CNS involvement varied among patients. ON was bilateral in four out of seven cases (patients 6, 7, 18, and 22) (57%). Myelitis involved every spinal segment, cervical, thoracic, or conus medullaris, either as longitudinally extensive transverse myelitis or as solitary lesions. A brain MRI, whenever executed, revealed white matter lesions in the brainstem (patient 12), cerebellum (patient 18), supratentorial and infratentorial white matter regions (patients 9, 10, and 22), and right frontal lobe and pons (patient 13). In other cases, it was reported as normal (patient 19). Interestingly, gadolinium enhancement was reported in most cases; cauda equina involvement, with gadolinium enhancement, was mentioned in most cases regardless of CNS involvement. Nevertheless, it was not observed in the two cases of brachial neuritis (patients 7 and 8), two cases with unilateral ON (UON) (patients 4 and 19), and in a case of myelitis (patient 17). 

Regarding demyelinating neuropathy, one was characterized as acute inflammatory demyelinating polyneuropathy (AIDP) (patient 3), two as MMN (patients 4 and 17), one as multifocal acquired demyelinating sensory and motor (MASDAM) (patient 9), two as brachial plexitis (patients 7 and 8), and two as radiculitis (patients 5, 6, 10, 12, and 14). The rest of the cases were characterized as demyelinating polyneuropathy CIDP.

CSF examination was included in the diagnostic workup in most cases; the most common finding wasrotein elevation. OCBs were not detected in the majority of cases, with the exception of four patients (7, 17, 18, and 22). In addition to MOG antibodies, the patients were screened for other antibodies, most commonly anti-AQP4, anti-contactin, anti-neurofascin 155, antiganglioside, anti-sulfatide, and paraneoplastic. All these tests yielded normal results with rare exceptions: anti-GM1 positive in patient 8 and anti-AQP4 in patient 18. 

Concerning immunotherapies, corticosteroids were used in all cases; sometimes followed by IVIG, plasma exchange (PE), and less often other immunosuppressive therapies such as azathioprine, rituximab, cyclophosphamide, methotrexate, and mycophenolate mofetil. In one case, spontaneous recovery was reported (patient 7). The response to therapy varied; some cases responded with complete resolution (patients 2, 4, and 5), while the rest showed improvement but with some deficits or relapses.

## 4. Discussion

This study reports four new cases of MOG-IgG seropositive demyelinating polyneuropathy adding to the few already published since 2018. In addition, a comprehensive review of the relevant literature was performed, summarizing all clinical, immunological, neurophysiological, and imaging findings, as well as treatment options and outcomes.

A wide range of ages were affected, from children to adults over 70 years old. No sex predominance was observed. Precedent events, such as infections, vaccination, or delivery were scarce. All cases had confirmed demyelinating polyneuropathy, following EFNS/PNS criteria. Two cases of brachial neuritis and five cases of radiculitis were included as focal, atypical CIDP. The clinical presentation varied among cases regarding CNS involvement, either ON and/or myelitis, as did the sequence of occurrence. In most cases, no other antibodies, apart from anti-MOG antibodies, were detected, capable of being possibly associated with demyelination. The CSF examination was unremarkable in most cases, except for elevated protein levels. The most common imaging finding was cauda equina involvement with gadolinium enhancement. Steroids were the treatment of choice, followed by a variety of other immunosuppressive therapies, in cases of incomplete response.

The potential mechanism of simultaneous PNS and CNS demyelination requires possibly antibodies against a common shared PNS and CNS epitope. It mainly involves autoantibodies against nodal and paranodal proteins, specifically anti-neurofascin (NF) antibodies [53] or against myelin antigens common to both the CNS and PNS, such as myelin-associated glycoprotein (MAG) [26]. NF isoforms are essential for assembling and stabilizing the nodal and paranodal domains of myelinated axons and therefore contribute to effective saltatory conduction [54]. The NF-155 isoform is expressed in glial cells in both the CNS and PNS, namely oligodendrocytes and Schwann cells [53]. Antibodies against the NF-155 isoform have been described in isolated CNS demyelinating disorders, such as (NMOSD) [53,55], in isolated PNS demyelinating disorders, such as AIDP and CIDP [56,57,58], and in CCPD [33,53]. The course of the disease varies, as does the response to treatment, sometimes reported as poor [25,58] and sometimes as favorable [33]. MAG, a transmembrane glycoprotein, is localized to the node of Ranvier in Schwann cells and oligodendrocytes and has been implicated in cases of CCPD [27,59].

AQP4 is a cell membrane water channel at the astrocyte foot processes of the blood–brain barrier and in the transition zone of the roots [27,60]. AQP4 antibody positivity in CCPD cases in cohort studies has been reported in a significant percentage, ranging from 21.0% to 37.5% [27,61], but not in other cohort studies [52]. Myelin basic proteins (MBPs) are a group of seven proteins, common in all myelin sheaths in the CNS and PNS, comprising 30% of the myelin sheath [62]. In the PNS it is referred to as P1. Although it could serve as a common target for CNS and PNS demyelination, to the best of our knowledge, no case of CCPD attributed to anti-MBP antibodies has been reported.

**Figure 1:**
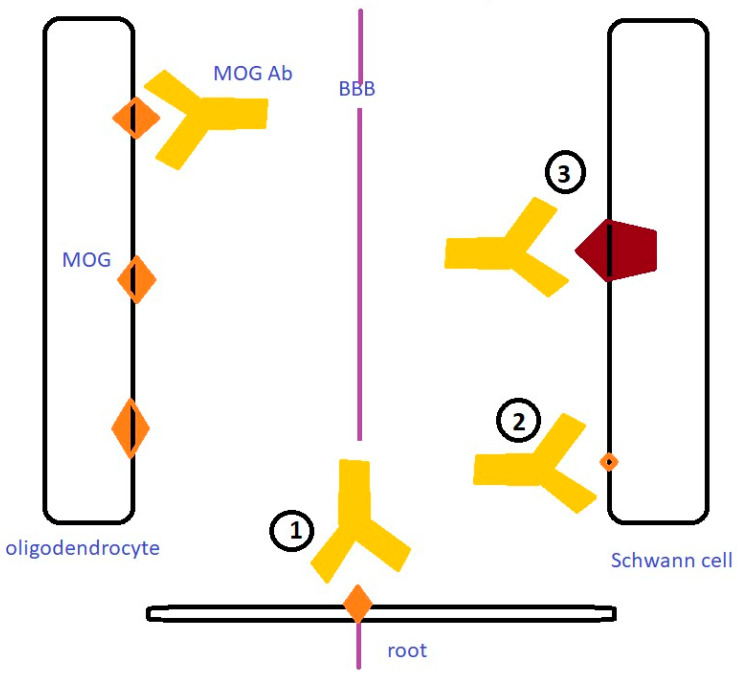
Possible mechanisms by which MOG-IgGs might attack peripheral myelin. 1. Roots are at the transition zone where CNS shifts to PNS; 2. Non-detectable MOG exists in Schwann cell; 3. MOG-IgGs recognize and attack other myelin antigens through mimicry.

MOG is detected on the outer membrane of myelin sheaths exclusively in the CNS [63]. The expression of MOG in peripheral nerves has been reported only in rats and not in humans [64]. Therefore, PNS involvement in cases of anti-MOG positivity is poorly understood. The coincidental co-occurrence of CNS and PNS involvement does not seem very likely, as new cases of CCPD attributed to MOG antibodies are constantly emerging. Furthermore, the partial or complete immunotherapy responsiveness could possibly indicate an inflammatory process of the PNS. The frequent cauda equina involvement might be explained by the proximity of roots to the CNS and an antibody leakage from the nerve root [49]; nevertheless, this hypothesis cannot explain cases in which isolated PNS involvement is noted. Other possible pathogenetic mechanisms might be the expression of very low quantities of MOG in Schwann cells, not detectable by the laboratory techniques used, but able to generate an immunological reaction [39]. Another hypothesis is that CNS inflammation might trigger a cascade of immune responses in the PNS targeting currently unidentified antigens in the nerve roots by means of molecular mimicry with peripheral myelin proteins [34]. Possible mechanisms by which MOG-IgGs might attack peripheral myelin are depicted in Figure 1.

CCPD is a rather heterogeneous disease and should not be considered as a simple coexistence of MS and CIDP. The diagnosis is based on fulfilling the criteria for CNS and PNS involvement and at the same time excludes secondary demyelinating diseases or changes, as defined by Ogata et al. [52]. In a recent study by Hou et al. [27], they enrolled 31 cases with CCPD, using strict inclusion and exclusion criteria. Patients were screened for several antibodies: AQP4, MOG, NF155, NF186, contactin 1 (CNTN1), CNTN2, contactin-associated protein-like 1 (CASPR1), CASPR2, MAG, and neuronal cell adhesion molecule (NrCAM). In their cohort study, none of the patients was positive for anti-MOG antibodies. They identified four anti-AQP4-positive, two NF-155, and one anti-MAG-positive patients. Otherwise, clinical, laboratory, neurophysiological, imaging, therapeutic, and responsiveness characteristics were common. The age range was wide, as reported by other studies. The CSF protein was elevated in 60% of patients, while OCB positivity was lower (36%). Spinal lesions were far more common than brain lesions, and abnormal VEPs were detected in 33.3%, while only 16.6% reported ON symptoms. Regarding PNS involvement, it was typical of a demyelinating process, like prolonged H-reflex latency and prolonged F-wave and prolonged distal latency. Most patients received corticosteroids and showed responsiveness. Other treatment options were tried in cases of partial resolution of symptoms, like rituximab, PE, and IFN-beta. Most cases showed a rather progressive course rather than a relapsing–remitting or monophasic course. In this cohort, a previous infection was reported by 1/3 of patients, a point of difference compared to the results of our review and in contrast to other reviews that reported lower (10%) [52] or higher rates (65%) [25]. Another difference is the reported higher prevalence in young adults and women, which was not seen in anti-MOG cases. 

In another observational retrospective study by Cortese et al. [25], 31 patients fulfilled the inclusion criteria; patients were screened for anti-AQP4, anti-gangliosides, and anti-sulfatide antibodies but not for anti-MOG antibodies. A wide age range was also reported (14–82), but with a male predominance. A previous infection or vaccination was commonly reported (65%), higher than in any other study. The PNS was primarily involved in 11 patients. Mixed signs of central and peripheral weakness were confirmed. Most patients showed a progressive or relapsing–remitting course rather than a monophasic course, and finally ended with a chronic progressive clinical course. In this cohort, the CSF protein was also elevated, and OCBs were rarely detected. In this study, brain and spinal lesions were described, as well as cauda equina involvement with contrast enhancement, as shown in MOGAD patients, but not in other CCPD cases [6]. Moreover, from the 31 included patients, only 23 (74%) fulfilled EFNS/PNS electrodiagnostic criteria for demyelinating neuropathy, like F-response latency prolongation and slow nerve conduction velocities and conduction blocks. It is argued that the remaining 26% of the cases characterized as axonal neuropathy may tend to represent later stages of the disease and that the axonal degeneration is a secondary phenomenon of the demyelination that preceded it. In most cases (71%), PNS and CNS involvement occurred simultaneously, as in anti-MOG-associated CCPD. VEPs were found prolonged in a significant proportion, even without clear symptoms of ON. The outcome was poor in the majority of the patients receiving either steroid or IVIG.

Lastly, in a nationwide survey in Japan [52], 40 patients were included. The age range was not as wide as in previous studies, ranging from 8 to 59 years. A female predominance was noted, in contrast to the Cortese et al. study. The clinical course was equally monophasic, relapsing, or chronic. Only 4/40 (10%) reported a history of previous infection and one after vaccination. Motor and sensory symptoms, of either central or peripheral origin, were observed in almost all patients, while OΝ was observed in 50% of patients. As described in the previous studies, the CSF protein was elevated, but OCBs were not positive except in a minority of cases (7.4%). Regarding antibodies, anti-AQP4 were not detected in any patient, whereas anti-NF-155 were positive in 5/11 cases (45%). Anti-MOG antibodies were not tested. Imaging studies revealed lesion load in both the brain and spine, some of them enhancing. No information is provided regarding cauda equina involvement. What is interesting in this study is the great response to steroids and IVIG treatment, in contrast to the other cohort studies, reaching a symptom resolution rate of 88.3%.

## 5. Conclusions

To the best of our knowledge, this is the first review of anti-MOG-positive cases with demyelinating polyneuropathy with or without CNS involvement. A high heterogeneity characterizes demographic and clinical features, as described in other CCPD cases, not related to anti-MOG antibodies. In all cases of CCPD, regardless of the specific antibody detected, the CSF findings, elevated protein, negative OCBs, higher age at onset, and above all, PNS involvement, distinguish CCPD from multiple sclerosis. The clinical course and response to treatment varied significantly in anti-MOG-positive cases as it did in other CCPD cases. 

Although rare, anti-MOG-associated CCPD should be considered in the differential diagnosis of demyelinating polyneuropathy, and CNS involvement should be persistently sought. The pathogenesis remains unclear, but standard treatment options, and especially steroids, remain the first-line therapeutic option, resulting in at least a partial resolution of symptoms even though most cases show relapses or a more chronic course.

Reporting new cases adds to the limited knowledge of this rare and devastating disease. Cumulative clinical, immunological, and laboratory data will allow a better characterization and understanding of the phenotype and underlying mechanisms, leading to early diagnosis and targeted treatments.

## Figures and Tables

**Table 1:** Demographic, clinical, neurophysiological, imaging, and immunological characteristics as well as treatment and response to treatment of the four new patients and the included studies.

		History	Sex	Age	PNS	ON	Myelitis	Sequence of Nervous System Involvement	Antibodies	CSF	MRI Features	Treatment	Comments
	Patient-A	History of ON	M	48	CIDP	−	+	Concurrent	Neurofascin-155 (−) Contactin-1 (−)	Normal OCB (+)	Brain Spine (C–T)	IVMP Interferon beta-1a AZA, MTX, RTX	Relapses
	Patient-B		M	67	CIDP	−	−	Concurrent	Gangliosides (−), Paraneoplasmatic (−)	Pr+ OCB (−)	Brain	IVMP improvement	Monophasic
	Patient-C		M	10	CIDP	UON	+	Concurrent	AQP4 (−) Gangliosides (−)	Pr+ Cells + OCB (−)	Brain Spine (C–T) Cauda equina	IVMP, IVIG RTX, MM	Relapses
	Patient-D		F	20	CIDP	−	+	Concurrent		PR+ OCBs+, (type II)	Brain Spine Gd+	IVMP Complete resolution	Relapses
1	Dinoto et al., 2022 [37]		M	55	CIDP	−	+	Myelitis first	Contactin (−) Neurofascin 155 (−)	Pr+ OCB NI	Spine (C)	No treatment	No MOG protein expression on peripheral nerve assessed by Western blot was observed Multifocal swellings in nerve US
2	Dinoto et al., 2022 [37]		F	74	CIDP	UON	−	ON preceded 10 MO	MOG (+) If 1>1/160 CBA Contactin (−) Neurofascin 155 (−)	Normal OCB (−)	NI	IVMP complete resolution	No MOG protein expression on peripheral nerve assessed by Western blot was observed
3	Rinaldi et al., 2021 [38]		F	9	AIDP	−	+	Concurrent	AQP4, NF186, CNTN1, CASPR1, LGI1, GQ1b, sulfatide (−)	OCB (−)	Spine (T3–T10) Conus Caude Equina Gd+	IVMP-IVIG incomplete resolution	Ongoing paraplegia thought to be secondary to spinal cord ischaemia /necrosis
4	Rinaldi et al., 2021 [38]		F	26	MMN	UON	−	PNS preceded 30 MO	GM1, GQ1b, NF186, CNTN1, CASPR1, LGI1, sulfatide (−)	not performed	Normal spinal	IVIG complete resolution	Relapsing Monthly IVIG
5	Rinaldi et al., 2021 [38]	3Ms Post-partum	F	31	Radiculitis	−	+	Concurrent	AQP4, NF186, CNTN1, CASPR1, LGI1, GQ1b, sulfatide (−)	Pr+ OCB (−)	Spinal T8 Conus Cauda equina Gd+	IVMP Complete resolution	Monophasic
6	Rinaldi et al., 2021 [38]		F	34	Radiculitis	BON	−	Concurrent	AQP4, NF186, CNTN1, CASPR1, LGI1, GQ1b, sulfatide (−)	Pr + OCB (−)	Spinal (C2, S1) cauda equina Gd+	IVMP, IVIG Complete resolution	Monophasic
7	Rinaldi et al., 2021 [38]		F	54	L Brachial neuritis	BON	−	ON first 23 MO	AQP4, NF186, CNTN1, CASPR1, LGI1, GQ1b, sulfatide (−)	OCBs (+) (serum and CSF)	NORMAL Spinal	Spontaneous recovery	Relapsing
8	Rinaldi et al., 2021 [38]	H1N1 vaccine 2Ws before	M	58	L Brachial neuritis	−	+	Brachial first 72 MO	AQP4 (−) GM1 (+)	OCBs (−)	Spinal T6–T10 Gd+	Steroids Partial resolution	Relapsing
9	Vasquez Do Campo et al., 2018 [34]	no preceding infection or vaccination	M	18	MADSAM	−	+	Concurrent over 3 W	AQP4, Contactin-1 Gngliosides, Sufatides, MAG, Paraneo (−)		Brain Spinal (C–T) Conus Cauda Equina Gd+	IVMP Clinical improvement-mild residuals	Sural biopsy: demyelinating MOG-IgG1 not detected after 9 M
10	Sundaram et al., 2019 [39]	DM	M	51	Radiculitis	−	+	Myelitis First 7 MO	AQP4 (−)	Pr+ OCBs (−)	Brain Spinal T12 Conus Cauda Equina Gd+	IVMP, AZA METH, RTX	relapsing
11	Shima and Tsujino, 2020 [40]	ON 20 Ys before	M	46	CIDP	UON	−	Concurrent	AQP4, Gangliosides, Paraneo Plasmatic Neurofascin 155 Contactin (−)	Pr+ OCB (−)	Cauda Equina	IVMP, PE steroids po Only ON improvement	No other CNS involvement
12	Nakamura et al., 2020 [41]	INFL A infection	M	40	Radiculitis	−	+	Concurrent		Pr+ OCBs (−)	Brain Spine C3-Conus Cauda Equina Gd+	IVMP, PE, IVIG, CP No clinical improvement	
13	Nakamura et al., 2021 [42]	3 MOs after normal delivery	F	32	CIDP	−	+	Concurrent	AQP4, Neurofascin 155, Ganglioside (−)	PR+ cells+ OCBs (−)	Brain Spinal C4, C7, T9) Cauda Equina Gd+	IVIG IVMP resolution	relapses PNS steroid response
14	Kang et al., 2021 [43]	No history infection or vaccination	M	72	Radiculitis	−	−	No CNS	Ganglioside, AQP4, MAG	CSF Normal	Cauda Equina Gd+	IVMP Improvement	
15	Kang et al., 2021 [43]	No infection or vaccination DM, HTN	M	46	CIDP	−	−	No CNS	AQP4 (−)	NI	Cauda Equina Gd+	IVMP	
16	Akbar et al., 2022 [44]	POST-COVID	F	9	CIDP	−	−	No CNS	Gangliosides (−)	Pr + OCBs (−)	Cauda Equina Gd+	IVIG (every 6–8 Ws) improvement	No CNS involvement
17	Elterefi et al., 2022 [45]		M	24	MMN	−	+	Concurrent	AQP4, Gangliosides (−)	Pr + OCBs (+)	Spinal (C–T) Gd +	IVIG, STEROIDS significant clinical improvement residual deficits	
18	Spiezia et al., 2022 [46]	NI	F	62	CIDP	BON	+	ON first PNS myelitis 4 MO later	AQP4 (+)	OCBs (+)	Brain Spinal C2–T1 Conus Cauda Equina Gd+	AZA, RTX Clinical improvement	Reduction in AQP4, MOG titres Improvement NCSs
19	Bosello et al., 2023 [47]	SARS-CoV-2 1M before DM, HTN	F	74	CIDP	UON	−	ON first CIDP 1 MO later	AQP4, Gangliosides (−)	normalL OCBs (−)	Normal MR brain/spine roots	STEROIDS IVIG improvement	Monophasic
20	Fuse et al., 2023 [48]	NI	M	48	CIDP	−	+	Concurrent	GM1, galactocerebroside (−)	NI	SpinailNI	STEROIDS PE Improvement	relapses
21	Bosisio et al., 2023 [47]	NI	F	7	CIDP	UON	NI	ON first 8 Ys			NI		Asynchronous
22	Horiguchi et al., 2024 [49]	six Ds after upper respiratory tract infection	F	10	CIDP	BON	+	Concurrent	AQP4, neurofascin 155, Contactin-1 Gangliosides (−)	Pr+ Cells + OCBs (+)	Brain Optic nerves Cauda Equina Gd +	IVMP improvement	Monophasic

AQP4: Aquaporin-4, AZA: azathioprine, BON: bilateral optic neuritis, CIDP: chronic inflammatory demyelinating polyneuropathy, CBA: cell-based assay, CBS: central nervous system, CP: cyclophosphamide, CSF: cerebrospinal fluid, D: days, DM: diabetes mellitus, F: female, Gd+: gadolinium enhancement, HTN: hypertension, IVIG: intravenous immunoglobulin, IVMP: intravenous methylprezolon, INFL: influenza, MADSAM: multifocal-acquired demyelinating sensory and motor, M: male, MOs: months, MTX: methorexate, MM: mycophenolate mofetil, MMN: multifocal motor neuropathy, NCSs: nerve conduction studies, NI: no information, OCBs: oligoclonal bands, ON: optic neuritis, PE: plasmapharesis, PN: polyneuropathy, PNS: peripheral nervous system, Pr: protein, RTX: rituximab, Spine (C–T–S): Spine (cervical–thoracic–sacral), UON: unilateral optic neuritis, US: ultrasound, Ys: years, Ws: weeks.

## Data Availability

All data are available from the corresponding author on reasonable request.

## Supplementary Materials

The following supporting information can be downloaded at https://www.mdpi.com/article/10.3390/jcm13123604/s1; Case reports appraisal; Case series appraisal. Figure S1: tiTimeline of the clinical case A; Figure S2: Timeline of the clinical case B; Figure S3: Timeline of the clinical case C; Figure S4: Timeline of the clinical case D.
