# Supplementary figures and images for: Combined Central and Peripheral Demyelination (CCPD) Associated with MOG Antibodies: Report of Four New Cases and Narrative Review of the Literature
Source: J Clin Med. 2024 Jun 20;13(12):3604. doi: 10.3390/jcm13123604 (PMC11204739; doi:10.3390/jcm13123604)

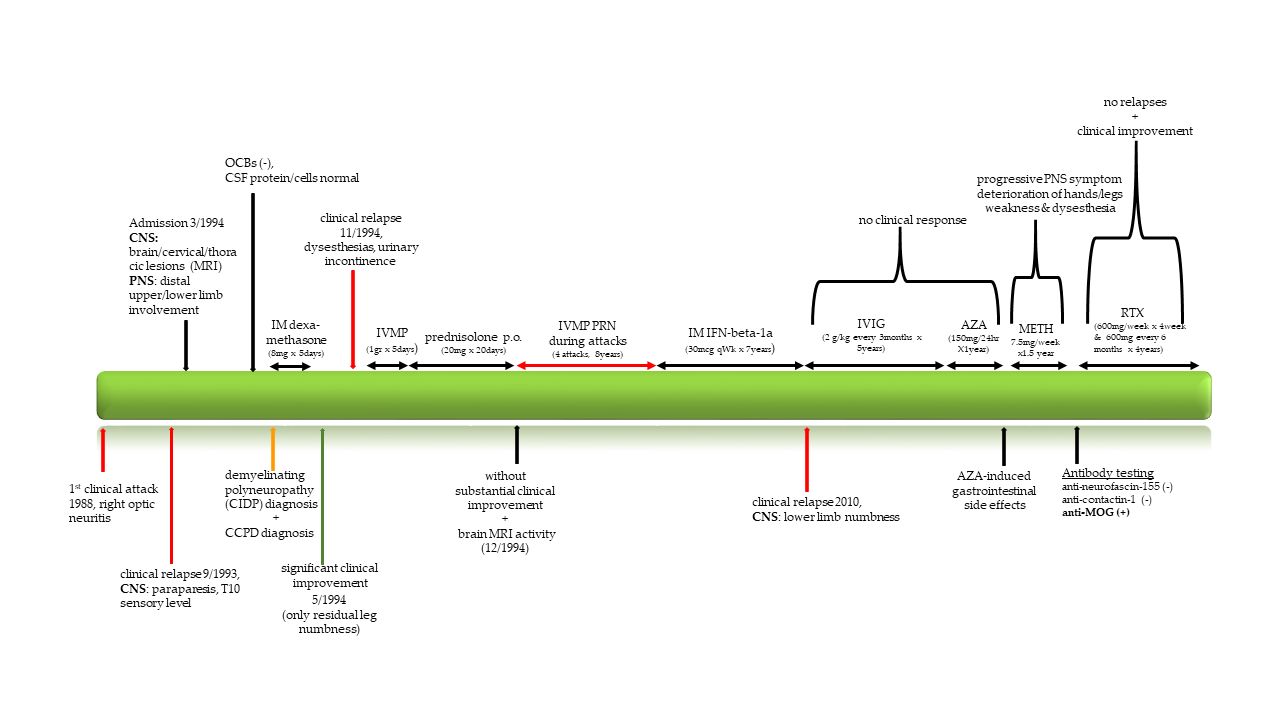

Supplement: Supplementary file 1 [file jcm-13-03604-s001.zip › Figure S1.TIF]

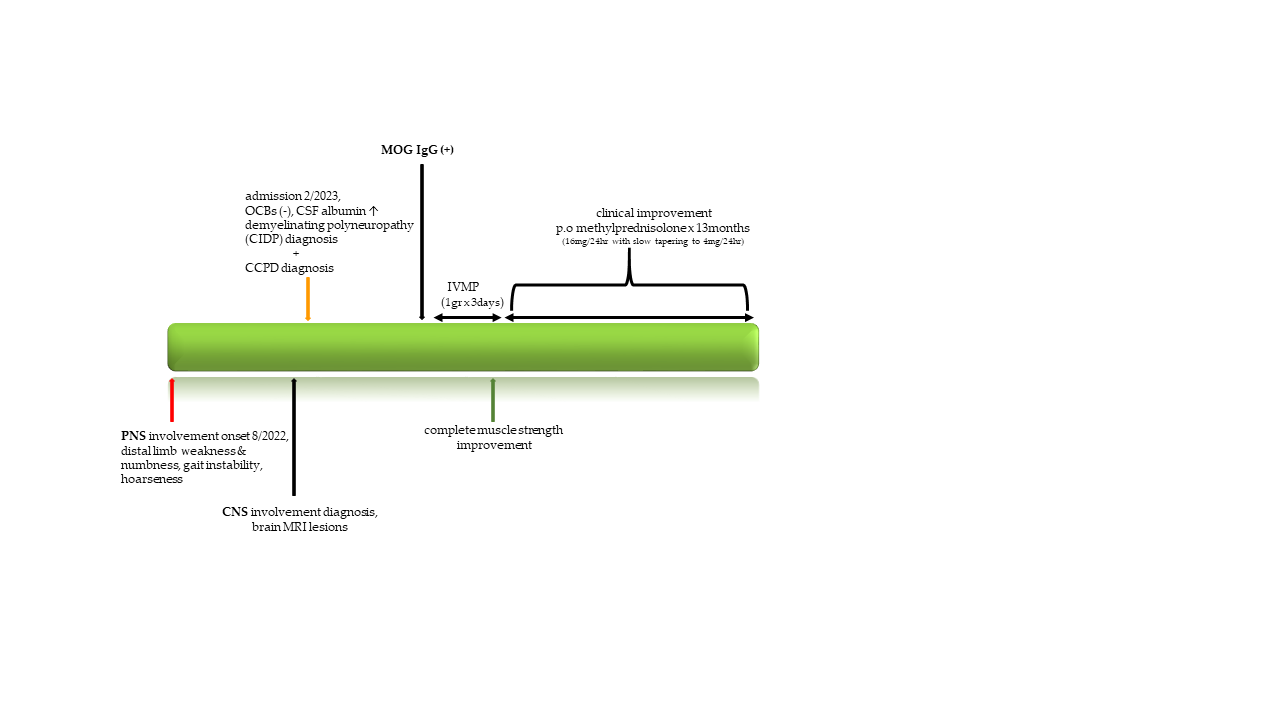

Supplement: Supplementary file 1 [file jcm-13-03604-s001.zip › Figure S2.TIF]

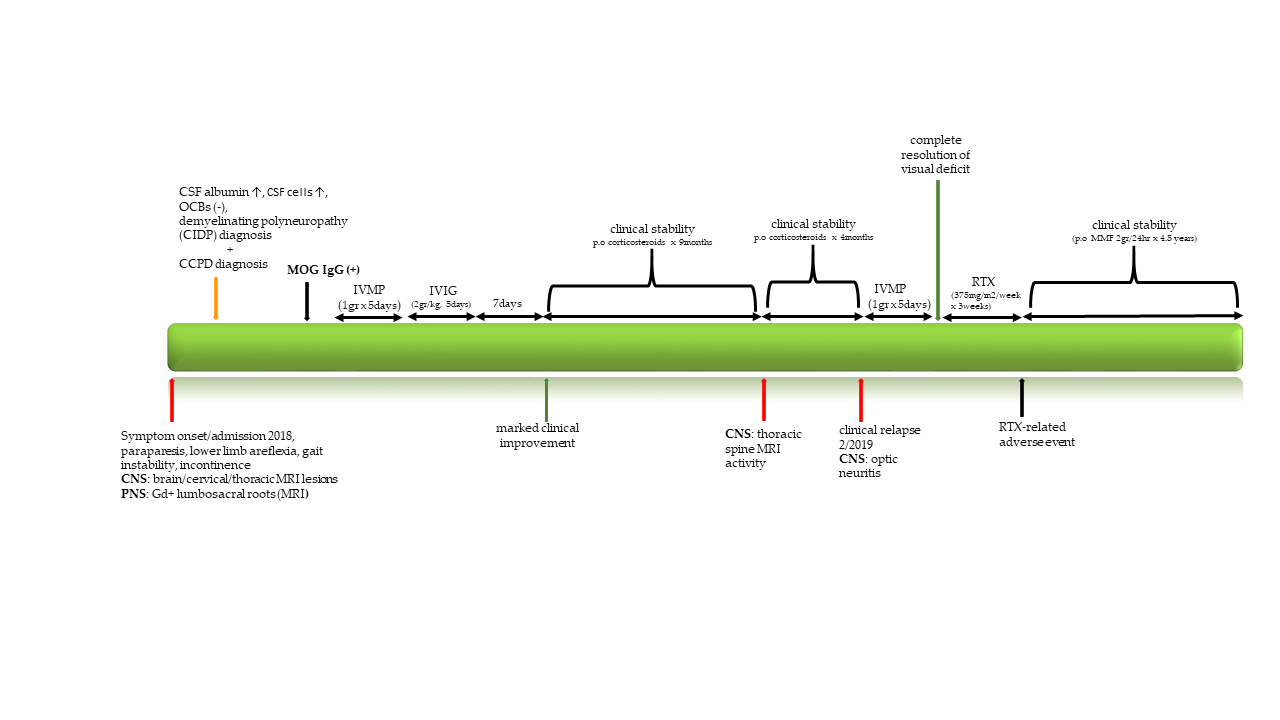

Supplement: Supplementary file 1 [file jcm-13-03604-s001.zip › Figure S3.TIF]

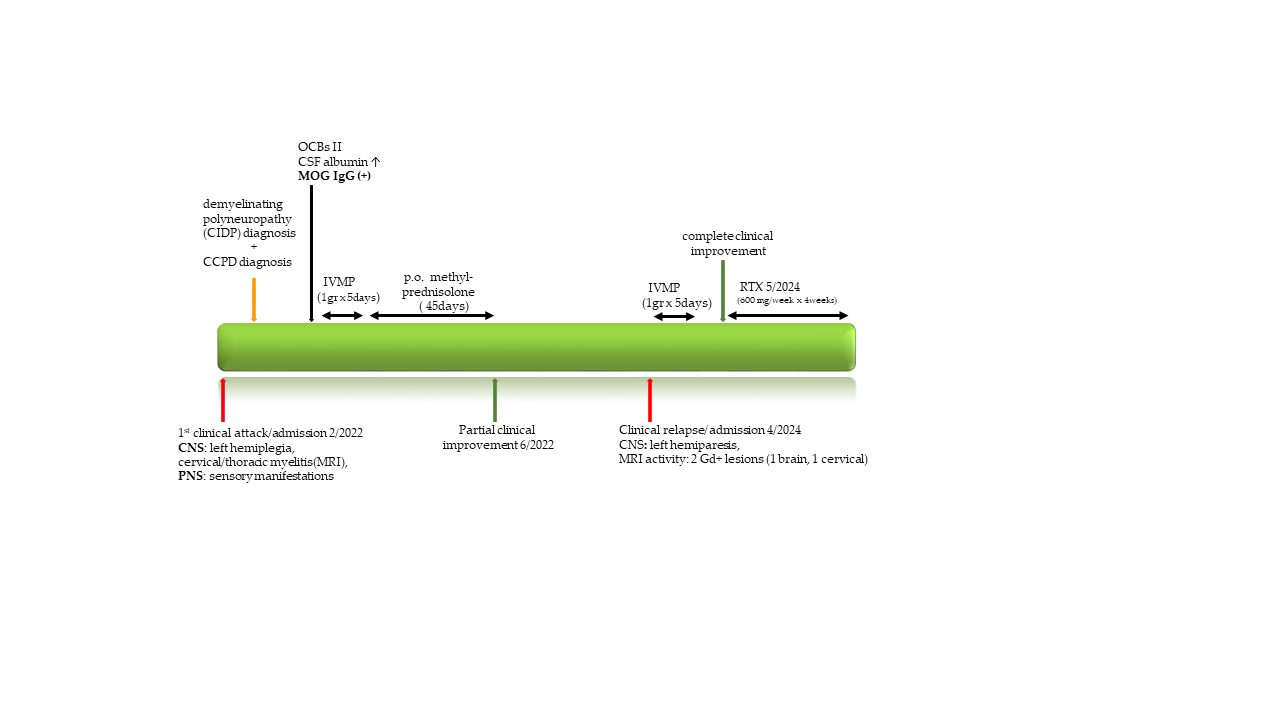

Supplement: Supplementary file 1 [file jcm-13-03604-s001.zip › Figure S4.TIF]
